# Cavernostomy x Resection for Pulmonary Aspergilloma: A 32-Year History

**DOI:** 10.1186/1749-8090-6-129

**Published:** 2011-10-05

**Authors:** Jorge MS Cesar, Jose S Resende, Nilson F Amaral, Carla MS Alves, Alyne F Vilhena, Frederico L Silva

**Affiliations:** 1 Department of Thoracic Surgery, Júlia Kubitschek Hospital, Fundação Hospitalar do Estado de Minas Gerais (FHEMIG), Belo Horizonte, Minas Gerais, Brazi

**Keywords:** Fungal infection, Haemoptysis, Lung surgery, Surgical management, Thoracic surgery, Tuberculosis

## Abstract

**Background:**

The most adequate surgical technique for the treatment of pulmonary aspergilloma is still controversial. This study compared two groups of patients submitted to cavernostomy and pulmonary parenchyma resection.

**Methods:**

Cases of pulmonary aspergilloma operated upon between 1979 and 2010 were analyzed retrospectively. Group 1 consisted of patients submitted to cavernostomy and group 2 of patients submitted to pulmonary parenchyma resection. The following variables were compared between groups: gender, age, number of hospitalizations, pre- and postoperative length of hospital stay, time of follow-up, location and type of aspergilloma, preoperative symptoms, underlying disease, type of fungus, preoperative pulmonary function, postoperative complications, patient progression, and associated diseases.

**Results:**

A total of 208 patients with pulmonary aspergilloma were studied (111 in group 1 and 97 in group 2). Group 1 was older than group 2. The number of hospitalizations, length of hospital stay and time of follow-up were higher in group 1. Hemoptysis was the most frequent preoperative symptom in group 1. Preoperative respiratory malfunction was more severe in group 1. Hemorrhagic complications and recurrence were more frequent in group 1 and infectious complications and residual pleural space were more common in group 2. Postoperative dyspnea was more frequent in group 2. Patient progression was similar in the two groups. No difference in the other factors was observed between groups.

**Conclusions:**

Older patients with severe preoperative respiratory malfunction and peripheral pulmonary aspergilloma should be submitted to cavernostomy. The remaining patients can be treated by pulmonary resection.

## Introduction

Controversies still exist regarding the most adequate surgical technique for the treatment of pulmonary aspergilloma despite decades of investigation. Some authors defend surgical treatment in all cases, even when asymptomatic, due to the risk of hemoptysis [1-11]. Other investigators indicate surgery only for symptomatic cases in view of the high rates of surgical morbidity and mortality [2,5,12-21]. Cavernostomy has only been indicated for the treatment of severely ill patients [5,17] whose clinical condition does not permit resection of the pulmonary parenchyma, or in cases of complex aspergillomas [1,5,6,18,22,23]. In contrast, Henderson and Pearson [16], Sagawa and colleagues [24] and Grima and colleagues [25] reported that cavernostomy is a simple and effective procedure which is free of complications and can be applied when more extensive surgery is contraindicated.

The present study evaluated factors associated with cavernostomy and pulmonary parenchyma resection, whose results will contribute to the choice of treatment of pulmonary aspergilloma.

## Patients and Methods

A retrospective study was conducted in which the records and radiologic exams of patients with pulmonary aspergilloma treated at the Julia Kubitschek Hospital between 1979 and 2010 were analyzed. The diagnosis of pulmonary aspergilloma was made based on clinical and radiologic examination, surgical findings, and isolation of the fungus. All cases were submitted to surgical treatment, irrespective of the presence of symptoms. The choice of the surgical technique was based on the location of the aspergilloma, pulmonary function, clinical condition of the patient, and preference of the surgeon.

The cases were divided into two groups: group 1 consisted of patients submitted to cavernostomy and group 2 of patients submitted to pulmonary parenchyma resection. The following variables were compared between groups: gender, age, number of hospitalizations, pre- and postoperative length of hospital stay, time of follow-up, location and type of aspergilloma, preoperative symptoms, underlying disease, type of fungus, preoperative pulmonary function, postoperative complications, patient progression, and associated diseases.

Peri- and postoperative bleeding, retained intrathoracic blood clots, and hemoptysis were classified as hemorrhagic complications. Infectious complications were septic shock, pleural empyema, and pulmonary infection. Pulmonary function was evaluated according to the classification of the American Thoracic Society [26]. Patients presenting recurrence and sequelae associated with treatment complications were classified as not cured. Cavernostomy was performed according to a previously described technique [18,27]. The fungus ball was localized by non-guided puncture during surgery. Two fragments of the costal arch were resected over the pulmonary cavern and a pneumotomy was performed over the lesion and kept opened with no suture. The fungus ball was removed with a spoon. The cavern was packed with gauze containing no antifungal substance. The gauze was removed after 24 h during cavernoscopy [27]. The cavity was left to close spontaneously and myoplasty was only performed when the cavity remained open for more than 90 days after surgery.

The Student *t*-test, chi-square test and Fisher's exact test were used for statistical analysis, with the level of significance set at 95%.

## Results

A total of 208 patients with pulmonary aspergilloma were studied, including 111 in group 1 and 97 in group 2. Group 1 was older than group 2, with mean ages of 42.13 and 37.67 years, respectively (*p *= 0.013). There were no gender differences between the two groups. Patients of group 1 were more frequently hospitalized (Table 1) and remained in the hospital for a longer period of time (Table 2) when compared to group 2. Follow-up was longer for patients of group 1 (1482.5 days) than group 2 (582.4 days) (*p *< 0.001).

**Table 1:** Number of Hospitalizations

Procedure	Number of hospitalizations^a^						
	
	Mean	Median	SD	Minimum	Maximum	25^th ^Percentile	75^th ^Percentile
Cavernostomy (n = 111)	1.8	1.0	1.2	1.0	6.0	1.0	2.0
Resection (n = 97)	1.4	1.0	0.9	1.0	7.0	1.0	1.0

SD: standard deviation.

^a ^*p *= 0.004 (*t*-test for equality of means, Levene's test for equality of variances).

**Table 2:** Length of Hospital Stay

Procedure	Length of hospital stay (days)^a^						
	
	Mean	Median	SD	Minimum	Maximum	25^th ^Percentile	75^th ^Percentile
Cavernostomy (n = 111)	128.8	90.0	114.0	3.0	540.0	41.0	200.0
Resection (n = 97)	59.3	30.0	67.9	7.0	450.0	16.0	71.0

SD: standard deviation.

^a ^*p *< 0.001 (*t*-test for equality of means, Levene's test for equality of variances).

Ten (9%) patients of group 1 required pulmonary parenchyma resection after cavernostomy because of recurrence. In group 2, lobectomy was performed on 53 patients (54.6%), pneumonectomy on 28 (28.9%), and segmentectomy on five (5.2%) (Table 3). There was no difference in the location (Table 4) or type of aspergilloma (Table 5) between the two groups.

**Table 3:** Pulmonary Parenchyma Resection

	Frequency	Valid percent
Bilobectomy	6	6.2
Lobectomy	53	54.6
Lobectomy + segmentectomy	5	5.2
Pneumonectomy	28	28.9
Segmentectomy	5	5.2
Total	97	100.0

**Table 4:** Location of Aspergilloma

			Location of aspergilloma				
			
			Bilateral	RSL	LSL	Others	Total
Procedure	Cavernostomy	No. of patients	4	54	41	12	111
		% within procedure	3.6%	48.6%	36.9%	10.8%	100.0%
	
	Resection	No. of patients	3	46	34	13	96
		% within procedure	3.1%	47.9%	35.4%	13.5%	100.0%

Total		No. of patients	7	100	75	25	207
		% within procedure	3.4%	48.3%	36.2%	12.1%	100.0%

LSL: left superior lobe; RSL: right superior lobe.

*p *= 0.942 (Pearson's chi-square). Number of valid cases = 207.

**Table 5:** Type of Aspergilloma

			Type of aspergilloma		
				
			Simple	Complex	Total
Procedure	Cavernostomy	No. of patients	11	95	106
		% within procedure	10.4%	89.6%	100.0%
	
	Resection	No. of patients	5	88	93
		% within procedure	5.4%	94.6%	100.0%

Total		No. of patients	16	183	199
		% within Procedure	8.0%	92.0%	100.0%

*p *= 0.196 (Pearson's chi-square test); *p *= 0.151 (Fisher's exact test). Number of valid cases = 199.

The frequency of preoperative symptoms was similar in groups 1 and 2, except for hemoptysis which was observed in 106 (95.5%) patients of group 1 and in 57 (58.8%) of group 2 (Table 6). There was no difference in the underlying disease between groups 1 and 2 (Table 7). Table 8 shows the type of fungus isolated from the aspergilloma.

**Table 6:** Preoperative Symptoms

Preoperative symptom	Group 1 (%)	Group 2 (%)	*p*
Cough	15 (13.5)	8 (8.2)	0.227
Hemoptysis	106 (95.5)	57 (58.8)	< 0.001
Fever	5 (4.5)	4 (4.1)	0.893
Weight loss	5 (4.5)	5 (5.2)	0.827
Chest pain	8 (7.2)	4 (4.1)	0.341
Dyspnea	11 (9.9)	5 (5.2)	0,199
Wheezing	5 (4.5)	4 (4.1)	0.893
Others	6 (5.4)	5 (5.2)	0.936

**Table 7:** Underlying Diseases

			Underlying disease					
				
			Pulmonary abscess	Bronchiectasis	Bronchogenic cyst	TBC	Others	Total
Procedure	Cavernostomy	No. of patients	2	2	2	101	0	107
		% within procedure	1.9%	1.9%	1.9%	94.4%	0.0%	100.0%
	
	Resection	No. of patients	2	1	2	87	3	95
		% within procedure	2.1%	1.1%	2.1%	91.6%	3.2%	100.0%

Total		No. of patients	4	3	4	188	3	202
		% within procedure	2.0%	1.5%	2.0%	93.1%	1.5%	100.0%

TBC: tuberculosis sequelae.

**Table 8:** Type of Fungus

			Type of fungus				
				
			*Aspergillus*	*Aspergillus fumigatus*	Fungus not identified	Others	Total
Procedure	Cavernostomy	No. of patients	15	37	19	6	77
		% within procedure	19.5%	48.1%	24.7%	7.8%	100.0%
	
	Resection	No. of patients	29	23	14	9	75
		% within procedure	38.7%	30.7%	18.7%	12,0%	100.0%

Total		No. of patients	44	60	33	15	152
		% within procedure	28.9%	39.5%	21.7%	9.9%	100.0%

*p *= 0.029 (Pearson's chi-square test); *p *= 0.028 (Fisher's exact test). Number of valid cases = 152.

Severe respiratory malfunction was more frequent in group 1 (n = 16, 27.1%) than in group 2 (n = 5, 7.7%). Normal respiratory function was observed in 23 (39%) patients of group 1 and in 34 (52.3%) of group 2 (*p *= 0.017).

Ten (10.3%) patients of group 2 and none of group 1 presented residual pleural space (*p *< 0.001). Dyspnea was more frequent in group 2 (n = 11, 11.3%) than in group 1 (n = 2, 1.8%) (*p *= 0.005). The incidence of fistulae did not differ between group 1 (n = 8, 7.2%) and group 2 (n = 7, 7.2%) (*p *= 0.602). Hemorrhagic complications were more frequent in group 1 (n = 50, 45%) than in group 2 (n = 12, 12.4%) (*p *< 0.001). Infectious complications were identified in eight (7.2%) patients of group 1 and in 24 (24.7%) of group 2 (*p *< 0.001). Recurrence was only observed in group 1 (n = 9, 8.1%) (*p *= 0.003).

Patient progression was similar in the two groups: 52 (67.5%) patients in group 1 were cured, 10 (13.0%) were not cured, and 15 (19.5%) died. In group 2, 56 (73.7%) patients were cured, 10 (13.2%) were not cured, and 10 (13.2%) died (*p *= 0.589).

The two groups were similar in terms of associated diseases: cardiovascular diseases were present in 14 (12.6%) patients of group 1 and in nine (9.3%) of group 2 (*p *= 0.295); diabetes in six (5.4%) patients of group 1 and in six (6.2%) of group 2 (*p *= 0.520); respiratory diseases in 15 (13.5%) patients of group 1 and in six (6.2%) of group 2 (*p *= 0.063), and other diseases in 21 (18.9%) patients of group 1 and in 13 (13.4%) of group 2 (*p *= 0.188).

### Comment

Patients of group 1 presented a longer mean hospital stay and a larger number of hospitalizations than those of group 2. This difference might be explained by the fact that most cavernostomies were performed at the beginning of the historical series when treatment was still institutional, including long periods of hospitalization. Another explanation is the larger number of patients with severe respiratory malfunction and of older patients in group 1. Kim and colleagues [28], Sagan and colleagues [11] and Sagawa colleagues [24] identified old age as a risk factor for postoperative complications. Oakley and colleagues [5] reported a mean length of hospital stay of 11 days for patients submitted to resection and of 16 days for those undergoing cavernostomy. The time of follow-up was significantly longer in group 1, probably because of the prolonged hospital stay of these patients. Henderson and colleagues [15] followed up patients for 2 to 48 months after surgery, with a mean of 20 months.

In the study of Oakley and colleagues [5], the main type of resection was right upper lobectomy, followed by left upper lobectomy. Right upper lobectomy was also the most common surgical procedure in other series [1,2,5,9,15,28]. Some investigators [5,18] recommend pneumonectomy to be avoided whenever possible because of postoperative complications. In the present series, lobectomy was the main type of resection. The large number of pneumonectomies (n = 28, 28.9%) and the small number of segmentectomies (n = 5, 5.2%) might be attributed to the predominance of complex aspergillomas and "destroyed lungs" due to tuberculosis sequelae.

In groups 1 and 2, aspergillomas were mainly found in the upper pulmonary lobes, with both lobes being affected in seven patients (3.4%). In the study of Chen and colleagues [2], most aspergillomas were located in the upper lobe (31 on the right and 28 on the left), a finding also reported in other studies [1,6,17,29,30]. This distribution might be associated with the presence of tuberculosis sequelae, hypoperfusion, or hypoventilation in the upper pulmonary segments [29]. Jewkes and colleagues [17] reported the presence of more than one aspergilloma in 19 (22%) of 85 patients, including 12 bilateral cases. Complex aspergillomas were observed in 95 (89.6%) patients of group 1 and in 88 (94.6%) of group 2, with no significant difference between groups. A higher frequency of complex aspergillomas has also been reported in other series [1,2,11,22,23,28].

Hemoptysis was the most frequent preoperative symptom in groups 1 and 2, and was observed in 163 patients (78.4%). Similar findings have been reported by other investigators [1,2,4,5,7-9,14,23,29,31,32]. However, hemoptysis was more frequent in group 1 than in group 2. This finding might be explained by the fact that most asymptomatic patients were allocated to group 2. In the study of Henderson and colleagues [15], cough with expectoration was the most frequent symptom, affecting 20 patients (83%). Hemoptysis was observed in 11 patients (46%). In the present series, the frequency of the other preoperative symptoms was similar in the two groups. Dyspnea and cough were the second most frequent symptoms after hemoptysis, in agreement with other studies [2,12,30-33].

Tuberculosis sequelae, but not active disease, were the underlying disease in 188 (93.1%) patients, with no difference between groups 1 and 2. Other studies also identified tuberculosis as the underlying disease [4,7-9,12,17,21,28,32]. Interestingly, sarcoidosis was not detected in any patient as the underlying disease, in contrast to other reports [2,33]. Tuberculosis and sarcoidosis were the most frequent underlying diseases in the studies of Battaglini and colleagues [1] and Nucci and colleagues [20]. The other underlying diseases cited by these authors have also been observed in our series, i.e., bronchiectasis, congenital cyst, and pulmonary abscess.

*Aspergillus fumigatus *was the fungus most frequently isolated from the pulmonary cavities of 37 (48.1%) patients in group 1 and *Aspergillus *sp was the most frequent fungus in group 2, detected in 29 patients (38.7%). Henderson and Pearson [16] found *A. fumigatus *to be the most common species in cultures of fungus balls.

Jewkes and colleagues [17] and Oakley and colleagues [5] used the presence of diffuse pulmonary disease and poor pulmonary function (forced expiratory volume in the first second (FEV_1_) < 1.34 liters and forced vital capacity (FVC) < 2.0 liters) as criteria for the indication of cavernostomy for the treatment of pulmonary aspergilloma. Cut-off values of mean predicted FVC of 78.5% and mean predicted FEV_1 _of 65% have been reported by Chen and colleagues [2] for the assessment of preoperative pulmonary function. In the present series, poor pulmonary function was observed in 16 (27.1%) patients of group 1 and in only five (7.7%) of group 2. The severity of preoperative respiratory malfunction may have been one criterion for the selection of patients for cavernostomy.

Hemorrhagic complications and recurrence were more frequent in group 1, and infectious complications and residual cavities were more common in group 2. Bleeding was the main postoperative complication in the study of Chen and colleagues [2]. According to Butz and colleagues [14], a post-resection pleural space is the most frequent complication after parenchyma resection for the treatment of aspergilloma. This residual pleural space may have been the cause of empyemas observed in group 2, especially when associated with a bronchopleural fistula as reported by other investigators [1,2,5,9,15,21,23,28]. In the present study, the incidence of fistulae was similar in the two groups, but the association of fistula, residual pleural space and tuberculosis sequelae was only observed in group 2. In addition, a fistula was present in seven (24%) of the 24 patients of group 2 with infectious complications, a fact explaining the higher incidence of infectious complications in this group. No fungal organism or bacterial superinfection was detected in any of the patients with pleural empyemas. The patients received no antifungal drugs, oral or intravenous triazoles [34] or amphotericin B as recommended by Sagan and colleagues [30]. No postoperative fungal dissemination was observed. A residual pleural space was a frequent postoperative finding in group 2 (n = 10, 10.3%), especially among patients with tuberculosis sequelae as the underlying disease. The latter has been considered to be a predisposing factor for complications and increased mortality [1,5,14]. Dyspnea was more frequent in group 2 despite better preoperative respiratory function. This finding can be explained by the fact that patients with diffuse pulmonary tuberculosis sequelae undergoing resection have no respiratory function reserve.

None of the present patients was submitted to arteriography or embolization for the treatment of significant hemoptysis because of the lack of a hemodynamic center in our hospital. However, embolization and CT-guided catheter drainage represent therapeutic alternatives for patients with pulmonary aspergilloma whose clinical condition does not permit surgery [35].

No significant difference in mortality was observed between groups 1 and 2. Mortality rates ranging from 1.1% to 44% have been reported in the literature [1,2,9,10,15,18,20,28]. This variation in mortality can be explained by the fact that surgery is performed on symptomatic and asymptomatic patients, with mortality being lower in the latter [18]. In the study of Jewkes and colleagues [17], three (7%) of 41 patients submitted to resection and four (44%) of nine patients submitted to cavernostomy with no clinical condition for major surgery died after the procedure. This difference was probably due to the fact that cavernostomy was reserved for more severely ill patients. Solit and colleagues [21] reported no elevated morbidity or mortality. Recent studies [2,4,5,8,10] have shown a significant reduction in surgical morbidity and mortality, encouraging a more frequent indication of surgery.

The evolution of patients in group 1 might be attributed to the selection of patients with more severe respiratory malfunction and of older patients. A prospective study could help clarify this issue. Both groups had their particular complications. Group 1 presented hemorrhagic complications and recurrence, which can be explained by the presence of residual pulmonary cavities after surgery. Group 2 more frequently developed infectious complications, associated with a residual pleural space, bronchopleural fistula and pulmonary tuberculosis sequelae, as discussed earlier. Advances in the surgical technique over the last years has permitted a more frequent indication of pulmonary resection, reserving cavernostomy to patients whose clinical condition does not permit resection surgery. The bronchial stump was not covered with viable tissue in any patient submitted to pulmonary resection because of the view that a residual pleural space is the main cause of empyema and that a bronchopleural fistula is a consequence rather than a cause. Prospective studies should be conducted to clarify this problem.

Groups 1 and 2 were similar in terms of associated diseases. Respiratory diseases were more frequent in group 1, with a difference of borderline significance between groups. A larger number of cases could confirm this difference between groups, showing that patients with respiratory disease were preferentially selected in group 1. Battaglini and colleagues [1] observed that more than half of 15 surgically treated patients had some other severe disease, a fact also reported by other investigators [21] and suggested that these associated medical problems contribute to mortality.

**Figure 1:**
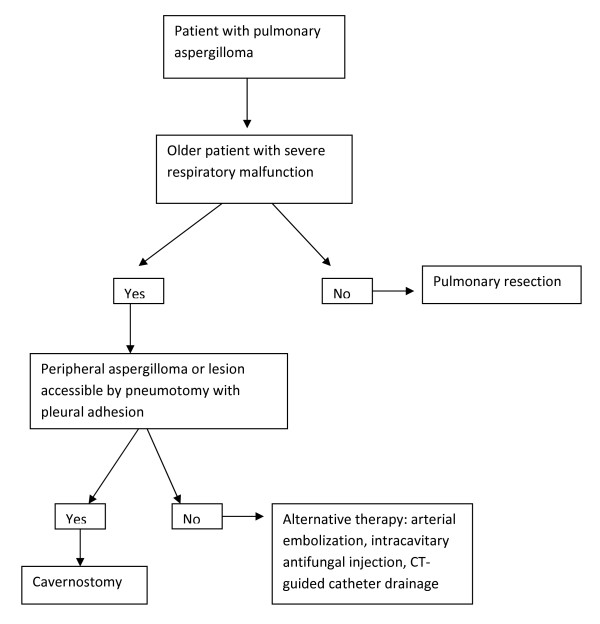
**Surgical therapeutic decisions in pulmonary aspergilloma**.

Preoperative respiratory function and age were the two factors differentiating groups 1 and 2 before surgical treatment. Since the two groups were similar in terms of postoperative progression, it seems reasonable to use these two variables to select the surgical therapy for pulmonary aspergilloma. Patients with central lesions not accessible by pneumotomy, with no pleural adhesions, and whose clinical condition does not permit pulmonary resection can be treated by arterial embolization, CT-guided catheter drainage, or intracavitary injection of antifungal drugs. However, these are only alternative therapies which have not been used in the present patients (Figure 1).

In conclusion, the present results suggest that the choice of surgical treatment for aspergilloma should be based on preoperative respiratory function and age. Older patients with severe preoperative respiratory malfunction and peripheral pulmonary aspergilloma, or lesions accessible by pneumotomy with pleural adhesions should be submitted to cavernostomy. Other patients with pulmonary aspergilloma can be treated by pulmonary resection.

## Competing interests

The authors declare that they have no competing interests.

## Authors' contributions

JMSC conceived of the study, and participated in its design and coordination. JSR participated in the design of the study. NFA participated in the design of the study. CMSA obtained data and drafted the manuscript. AFV obtained data and performed the statistical analysis. FLS obtained data and drafted the manuscript. All authors read and approved the final manuscript.

## References

[B1] Battaglini JW Murry GF Keagy BA Surgical management of symptomatic pulmonary aspergilloma Ann Thorac Surg 1985 39 512 6 10.1016/S0003-4975(10)61986-8 3890782

[B2] Chen J Chang Y Luh S Surgical treatment for pulmonary aspergilloma: a 28 year experience Thorax 1997 52 810 3 10.1136/thx.52.9.810 9371213 PMC1758650

[B3] Gilli SCO Gilbert MMP Frazatto C Jr Tratamento cirúrgico de "bola fúngica" em leucemia linfóide aguda (LLA) Rev Ass Med Brasil 1992 36 174 6 1340369

[B4] Kay PH Surgical management of pulmonary aspergilloma Thorax 1997 52 753 4 10.1136/thx.52.9.753 9371201 PMC1758649

[B5] Oakley RE Petrou M Goldstraw P Indications and outcome of surgery for pulmonary aspergilloma Thorax 1997 52 813 5 10.1136/thx.52.9.813 9371214 PMC1758638

[B6] Shapiro MJ Albelda SM Mayock RL Severe hemoptysis associated with pulmonary aspergilloma. Percutaneous intracavitary treatment Chest 1988 94 1225 31 10.1378/chest.94.6.1225 3191764

[B7] Shirakusa T Ueda H Saito T Surgical treatment of pulmonary aspergilloma and *Aspergillus *empyema Ann Thorac Surg 1989 48 779 82 10.1016/0003-4975(89)90670-X 2688579

[B8] Park CK Jheon S Results of surgical treatment for pulmonary aspergilloma Eur J Cardiothorac Surg 2002 21 918 23 10.1016/S1010-7940(02)00104-5 12062287

[B9] Caidi M Kabiri H Al Aziz S El Malout A Benosman A Surgical treatment of pulmonary aspergilloma. 278 cases Presse Med 2006 35 1819 24 10.1016/S0755-4982(06)74907-7 17159733

[B10] Lee JG Lee CY Park IK Pulmonary aspergilloma: analysis of prognosis in relation to symptoms and treatment J Thorac Cardiovasc Surg 2009 138 820 5 10.1016/j.jtcvs.2009.01.019 19660294

[B11] Sagan D Gozdziuk K Korobowicz E Predictive and prognostic value of preoperative symptoms in the surgical treatment of pulmonary aspergilloma J Surg Res 2010 163 35 43 10.1016/j.jss.2010.03.012 20850649

[B12] Addrizzo-Harris DJ Harkin TJ McGuinness G Pulmonary aspergilloma and AIDS. A comparison of HIV-infected and HIV-negative individuals Chest 1997 111 612 8 10.1378/chest.111.3.612 9118696

[B13] Allan A Sethia B Turner MA Recent experience of the treatment of aspergilloma with a surgical stapling device Thorax 1986 41 483 4 10.1136/thx.41.6.483 3787524 PMC460370

[B14] Butz OR Zvetina JR Leininger BJ Ten-year experience with mycetomas in patients with pulmonary tuberculosis Chest 1985 87 356 8 10.1378/chest.87.3.356 3971761

[B15] Henderson AH Deslaurier J Ritcey EL Surgery in pulmonary aspergillosis J Thorac Cardiovasc Surg 1975 70 1088 94 1186285

[B16] Henderson AH Pearson JEG Treatment of bronchopulmonary aspergillosis with observations on the use of natamycin Thorax 1968 23 519 23 10.1136/thx.23.5.519 5303008 PMC471841

[B17] Jewkes J Kay PH Paneth M Pulmonary aspergilloma: analysis of prognosis in relation to haemoptysis and survey of treatment Thorax 1983 38 572 8 10.1136/thx.38.8.572 6612647 PMC459613

[B18] Loeckell H On the transthoracic evacuation of a pulmonary mycetoma using Maurer drainage Prax Pneumol 1964 18 757 62 14334467

[B19] Niwa H Yamakawa Y Fukai I Subclavian artery branch ligation reduces hemorrhage during resection of pulmonary aspergilloma Ann Thorac Surg 1995 59 1234 5 10.1016/0003-4975(94)00850-7 7733734

[B20] Nucci M Pulcheri W Spector N Tratamento de micetoma pulmonar em pacientes neutropênicos Rev Ass Med Brasil 1993 39 188 8281206

[B21] Solit RW McKeown JJ Jr Smullens S The surgical implications of intracavitary mycetomas (fungus balls) J Thorac Cardiovasc Surg 1971 62 411 22 5126682

[B22] Belcher JR Plummer NS Surgery in broncho-pulmonary aspergillosis Br J Dis Chest 1960 54 335 41 10.1016/S0007-0971(60)80067-8

[B23] Daly RC Pairolero PC Piehler JM Pulmonary aspergilloma. Results of surgical treatment J Thorac Cardiovasc Surg 1986 92 981 98 3097424

[B24] Sagawa M Sakuma T Isobe T Cavernoscopic removal of a fungus ball for pulmonary complex aspergilloma Ann Thorac Surg 2004 78 1846 8 10.1016/j.athoracsur.2003.07.021 15511496

[B25] Grima R Krassas A Bagan P Badia A Le Pimpec Barthes F Riquet M Treatment of complicated pulmonary aspergillomas with cavernostomy and muscle flap: interest of concomitant limited thoracoplasty Eur J Cardiothorac Surg 2009 36 910 3 10.1016/j.ejcts.2009.05.007 19595606

[B26] American Thoracic Society. Medical Section of the American Lung Association Lung Function Testing Am Rev Respir Dis 1991 144 1202 18 1952453 10.1164/ajrccm/144.5.1202

[B27] Maurer G Cavernostomy and tamponade of pulmonary cavities with para aminosalicylic acid Dis Chest 1949 16 676 80 10.1378/chest.16.6.676 15396515

[B28] Kim YT Kang MC Sung SW Kim JH Good long-term outcomes after surgical treatment of simple and complex pulmonary aspergilloma Ann Thorac Surg 2005 79 294 8 10.1016/j.athoracsur.2004.05.050 15620961

[B29] Henderson AH Allergic aspergillosis: review of 32 cases Thorax 1968 23 501 12 10.1136/thx.23.5.501 5680235 PMC471837

[B30] Sagan D Gozdziuk K Surgery for pulmonary aspergilloma in immunocompetent patients: no benefit from adjuvant antifungal pharmacotherapy Ann Thorac Surg 2010 89 1603 10 10.1016/j.athoracsur.2010.02.037 20417786

[B31] Greenberg AK Knapp J Rom WN Clinical presentation of pulmonary mycetoma in HIV-infected patients Chest 2002 122 886 92 10.1378/chest.122.3.886 12226028

[B32] Ruiz Júnior RL de Oliveira FH Piotto BL Muniz FA Cataneo DC Cataneo AJ Surgical treatment of pulmonary aspergilloma J Bras Pneumol 2010 36 779 83 10.1590/S1806-37132010000600016 21225182

[B33] Mariotta S Giuffreda E Tramontano F Therapeutic approach in pulmonary mycetoma Panminerva Med 2001 43 161 5 11579328

[B34] Walsh TJ Anaissie EJ Denning DW Treatment of aspergillosis: clinical practice guidelines of the infectious diseases society of America CID 2008 46 327 60 10.1086/525258 18177225

[B35] Judson MA Stevens DA The treatment of pulmonary aspergilloma Curr Opin Invest Drugs 2001 2 1375 7 11890350

